# Common Duckweed (*Lemna minor*) Is a Versatile High-Throughput Infection Model For the *Burkholderia cepacia* Complex and Other Pathogenic Bacteria

**DOI:** 10.1371/journal.pone.0080102

**Published:** 2013-11-06

**Authors:** Euan L. S. Thomson, Jonathan J. Dennis

**Affiliations:** Department of Biological Sciences, University of Alberta, Edmonton, Alberta, Canada; Ghent University, Belgium

## Abstract

Members of the *Burkholderia cepacia* complex (Bcc) have emerged in recent decades as problematic pulmonary pathogens of cystic fibrosis (CF) patients, with severe infections progressing to acute necrotizing pneumonia and sepsis. This study presents evidence that *Lemna minor* (Common duckweed) is useful as a plant model for the Bcc infectious process, and has potential as a model system for bacterial pathogenesis in general. To investigate the relationship between Bcc virulence in duckweed and *Galleria mellonella* (Greater wax moth) larvae, a previously established Bcc infection model, a duckweed survival assay was developed and used to determine LD_50_ values. A strong correlation (R^2^ = 0.81) was found between the strains’ virulence ranks in the two infection models, suggesting conserved pathways in these vastly different hosts. To broaden the application of the duckweed model, enteropathogenic *Escherichia coli* (EPEC) and five isogenic mutants with previously established LD_50_ values in the larval model were tested against duckweed, and a strong correlation (R^2^ = 0.93) was found between their raw LD_50_ values. Potential virulence factors in *B. cenocepacia* K56-2 were identified using a high-throughput screen against single duckweed plants. In addition to the previously characterized antifungal compound (AFC) cluster genes, several uncharacterized genes were discovered including a novel *lysR* regulator, a histidine biosynthesis gene *hisG*, and a gene located near the gene encoding the recently characterized virulence factor SuhB_*Bc*_. Finally, to demonstrate the utility of this model in therapeutic applications, duckweed was rescued from Bcc infection by treating with bacteriophage at 6-h intervals. It was observed that phage application became ineffective at a timepoint that coincided with a sharp increase in bacterial invasion of plant tissue. These results indicate that common duckweed can serve as an effective infection model for the investigation of bacterial virulence factors and therapeutic strategies to combat them.

## Introduction

 Since their initial characterization as the causative agents of sour skin onion rot [1], members of the *Burkholderia cepacia* complex (Bcc) have gained particular notoriety as important opportunistic airway pathogens of cystic fibrosis (CF) patients [2]. CF patients are characteristically defective in mucus clearance, which creates ideal growth conditions for bacteria, particularly those able to withstand the prolonged acute inflammatory conditions present in the CF lung, including the Bcc [3]. The damage to lung tissues that results from such inflammation further promotes colonization by Bcc. The combination of defective mucus clearance and tissue damage result in significant degradation of lung cellular structure and pulmonary function that, in severe cases, can lead to “*cepacia* syndrome”, characterized by invasive necrotizing pneumonia and sepsis [4]. Bcc bacteria display exquisite innate and acquired resistance to antibiotic treatment [5-7] and Bcc infections are becoming increasingly widespread, thus creating a need for the development of alternative treatments and an increased understanding of the pathogenesis of these versatile Gram-negative bacteria. 

A number of animal infection models have been adapted for Bcc infection studies, such as chronic pulmonary infection-mimicking agar bead models for mice and rats [8-12]. In addition, a number of alternative animal infection models have been used with the Bcc, including *Galleria mellonella* (Greater wax moth) larvae [13]; *Drosophila melanogaster* (Common fruit fly) [14]; *Caenorhabditis elegans* (nematode) [15]; and *Danio rerio* (zebrafish) embryos [16]. In wax moth larval and murine infection models, bacteriophage therapy has been demonstrated as a potential means of circumventing the antibiotic resistance problems commonly associated with Bcc infection [17,18]. 

Because of similarities in cell and tissue types, innate and adaptive immune systems, and metabolism, animals intuitively represent a more accurate model than plants for the depiction of the human infection process and evaluation of alternative therapies. Even so, plants are gaining recognition for their usefulness in modeling bacterial pathogenesis in animal hosts, in some cases revealing conserved infection mechanisms [8,19-22]. Plants have innate immune systems that respond to invading bacteria, viruses and fungi [23] with the production of oxidative bursts, secondary metabolites and antimicrobial peptides [24-26], offering parallels to some of the most important stresses that pathogens encounter in animal hosts. For bacterial pathogens that are able to infect a wide range of hosts, including many Bcc species, plants represent inexpensive and easily manipulated models for the exploration of virulence factors, the infection process, and the evolutionary processes through which bacteria emerge as human pathogens. Plant models that have been used previously to study the Bcc include an onion maceration model [27], a simple alfalfa infection model [8], and a pea rhizosphere colonization model [28]. For other bacterial pathogens able to infect a wide range of hosts, such as *Pseudomonas aeruginosa*, the utilization of multiple infection models has led to the identification of several common virulence factors [29]. However, this same multi-host approach has not yet resulted in the discovery of many shared, universal virulence genes for the bacterial species of the Bcc [30]. Therefore, there remains a need for yet another versatile alternative infection model for the identification and study of virulence factors active during Bcc pathogenesis. 

 Common duckweed (*Lemna minor*) is one of the smallest known flowering plants and can be found growing on the surfaces of freshwater bodies throughout the world. These monocotyledon plants reproduce both sexually via flower fertilization and asexually by budding, the latter strategy providing a means by which to generate a large clonal population from a single plant. By eliminating the genetic variability common to current plant and animal infection models, the duckweed model allows the infection process to be studied with greater reproducibility. Axenic or sterile cultures of duckweed are easily obtained, thereby permitting the examination of an isolated, bipartite bacterial infection process. To date, several human pathogens have been studied using this model system [31]. For example, chromosomal disruption of the *lasIR* and *rhlIR* quorum sensing in *P. aeruginosa* eliminated bacterial inhibition of duckweed, indicating that the tissue-degrading proteases and other virulence factors known to be governed by these systems may have malignant effects on the plants as well. In the same study, the clinically relevant *Staphylococcus aureus* RN4220 strain was compared with attenuated strain ATCC 25923; RN4220 was found to exhibit extremely high virulence against the plants, whereas ATCC 25923 had no observable effect. Enterohaemorrhagic Escherichia coli 0157:H7 also displayed enhanced virulence relative to attenuated *E. coli* strain DH5α [31]. 

 In this study, we investigated the potential of duckweed as a Bcc infection model by several different approaches. First, we introduce a 50% lethal dose (LD_50_) -based virulence experiment and utilize this approach to show a quantitative relationship between LD_50_ values obtained with a panel of Bcc strains in the *L. minor* model versus the established *G. mellonella* model. A possible mechanism of plant killing is identified using lyophilized supernatants of *Burkholderia cenocepacia* strain K56-2 and a *shvR* mutant obtained in this screen that is deficient in the production of a known phytotoxin. These results prompted us to employ the duckweed infection model with other human bacterial pathogens, and we show that a strong relationship between plant and insect pathogenesis exists for a panel of enteropathogenic *E. coli* (EPEC) mutants. The high-throughput potential of the duckweed model is demonstrated by performing a virulence factor screen of a *B. cenocepacia* K56-2 mutant library, resulting in the identification of several novel putative virulence factors. To demonstrate the potential for this model in therapeutic investigations, bacteriophage rescue of *B. cenocepacia*-infected plants is demonstrated. Treatment before 12 h is required for plant rescue, and bacterial escape into plant tissues is demonstrated as a mechanism for this limitation. 

## Materials and Methods

### Reagents, strains, plasmids, antibiotics and culture conditions

 Unless otherwise specified, all reagents were obtained from Sigma-Aldrich Canada (Oakville, ON), and all media were obtained from Difco Laboratories (Detroit, MI). Bacterial strains used in this study are listed in Table 1. Overnight bacterial cultures were carried out in 2 mL ½ Luria-Bertani (½ LB) broth in 15-mL conical tubes (VWR International, Radnor, PA) for 18 h at 30°C with shaking at 225 rpm, unless otherwise noted. Antibiotics were included at the following concentrations where noted: tetracycline (Tc), 10 µg/ml for *E. coli* and 100 µg/ml for *B. cenocepacia*; trimethoprim (Tp), 100 µg/ml.

**Table 1:** Bacterial strains and species used in this study, including LD_50_ values.

**Species**	**Strain**	**Source or relevant genotype or phenotype^a^**	**LD_50_ in *L. minor* (cfu/ml) +/- SE^b^**	**LD_50_ in *G. mellonella* (cfu)**
*Burkholderia cepacia*	LMG 18821	CF, Australia	1.9x10^2^ +/- 1.2x10^2^	3.0x101 [13]
	LMG 2161	Soil, Trinidad	2.5x10° +/- 5.3x10^-1^	1.0x100 [13]
*B. multivorans*	C5393	CF, Canada	>1.0x10^9^	>3.0x106 [13]
	C3430	CF, Canada	>1.0x10^9^	>3.0x106 [13]
	C5274	CF, Canada	3.1x10^9^ +/- 2.4x10^9^	1.0x106 [13]
	C5568	CF, Canada	1.1x10^8^ +/- 8.8x10^6^	>3.0x106 [13]
*B. cenocepacia*	PC715j	CF, Canada	5.0x10° +/- 5.0x10^-1^	4.0x103 [13]
	J2315	CF-e, UK	1.6x10^6^ +/- 7x10^5^	1.0x105 [13]
	K56-2	CF-e, Canada	1.2x10^1^ +/- 7.0x10°	9.0x102 [13]
	C1257	CF, Canada	8.8x10° +/- 5.7x10°	4.0x104 [13]
	C4455	CF, Canada	1.01x10^5^ +/- 3.9x10^4^	1.0x105 [13]
	C5424	CF, Canada	5.3x10^3^ +/- 4.5x10^3^	2.0x105 [13]
	C6433	CF, Canada	2.8x10^4^ +/- 2.2x10^4^	3.0x104 [13]
	Cep511	CF, Australia	7.6x10° +/- 3.4x10°	8.0x108 [13]
	K56-2 *shvR*::Tp^R^	Plasposon insertion into *bcas0225*	>3.0x10^8^	100% virulence (i.e., ~9.0x102) [30]
*B. stabilis*	LMG 14294	CF, Belgium	>1.0x10^9^	2.0x106 [13]
	LMG 18870	CF, Canada	>1.0x10^9^	>2.0x106 [13]
*B. vietnamiensis*	DBO1	Soil, United States	3.7x10^2^ +/- 2.5x10^2^	2.0x105 [13]
	PC259	CF, United States	>1.0x10^9^	>3.0x106 [13]
*B. dolosa*	AU0645	CF, United States	>1.0x10^10^	>4.0x106 [13]
	STM1441	Rhizosphere, Senegal	4.8x10^7^ +/- 1.3x10^7^	4.0x104 [13]
*B. ambifaria*	Cep0996	CF, Australia	5.8x10^8^ +/- 4.4x10^8^	8.0x105 [13]
	AMMD	Rhizosphere, United States	2.5x10° +/- 4.0x10^-1^	n.d.
*B. anthina*	J2552	Rhizosphere, UK	4.5x10^8^ +/- 5.2x10^7^	3.0x105 [13]
*B. pyrrocinia*	ATCC 15958	Soil, Japan	2.5x10° +/- 7.9x10^-1^	3.0x102 [13]
*Acinetobacter baumanii*	AYE	Clinical, France	>1.0x10^9^	2.01x105 [52]
	ACICU	Clinical, Italy	>1.0x10^9^	5.58x105 [52]
	ATCC 17978	Clinical, France	>1.0x10^9^	2.42x105 [52]
	SDF	Human body louse	>1.0x10^9^	4.84x107 [52]
*Escherichia coli*	E2348/69	Parent strain	9.0x10^1^ +/- 3.8x10^1^	2.57x103 [37]
	JPN15	E2348/69 cured of EAF plasmid	7.9x10^4^ +/- 3.0x10^4^	1.2x108 [37]
	*ΔescN*	E2348/69 deficient in type III secretion	6.9x10^2^ +/- 4.7x10^2^	1.82x105 [37]
	*ΔbfpA*	E2348/69 deficient in bundle forming pilus	6.0x10^2^ +/- 5.9x10^2^	4.9x103 [37]
	*ΔcpxR*	E2348/69 deficient in Cpx pathway activation	1.0x10^2^ +/- 9.7x10^1^	4.17x104 [37]
	cpxA24*	E2348/69 with constitutive ON Cpx pathway	6.3x10^5^ +/- 4.9x10^5^	2.5x1010 [37]
	*perA*::Km^R^	E2348/69 deficient in type III secretion and bundle forming pilus	<1.0x10^3^	n.d.
*Campylobacter jejuni*	11168	Feces of diarrheic patient	>1.0x10^9^	n.d. [47]
	81-176	Feces of diarrheic patient	>1.0x10^9^	n.d. [47]
*Ralstonia solanacearum*	GMI1000	Tomato plant	>1.0x10^9^	n.d.

a Abbreviations: CF, cystic fibrosis infection; CF-e, strain that has spread epidemically among CF patient; n.d., not determined.

b S.E., standard error of the mean, included where applicable

### Duckweed growth and sterilization

 Duckweed plants were obtained from the greenhouse in the Biological Sciences building of the University of Alberta. To sterilize the plant surfaces for axenic growth, plants were submerged in 10% v/v bleach for 10 s, transferred into 70% v/v ethanol for 10 s, then transferred into sterile Schenk-Hildebrandt medium supplemented with 1% w/v sucrose (SHS) to recover. Plants were grown statically in 24-well plates at 30°C, as previously described [31]. Maintaining the plants under a light/dark cycle of 18/6 h promotes asexual reproduction by division, and under these conditions plants undergo three to four generations per week. 

### Duckweed infection

 Each well of a 96-well plate was filled with 180 μl of SHS and one duckweed plant comprising 2-3 fronds (i.e., at an intermediate stage of its growth). One millilitre of an overnight bacterial culture was centrifuged for 5 min at 5,000 × *g*, resuspended in 1 ml SHS to wash the cells, centrifuged again and then resuspended in a final volume of 1 ml SHS. For strains exhibiting higher lethality, cells were then diluted in SHS to an appropriate concentration for the infection. For 10-fold serial dilutions, twenty microlitres of the final cell suspension were added to the first column of eight wells in a 96-well plate and serially diluted using a Research multichannel micropipettor (Eppendorf, Hamburg, Germany), leaving a final volume of 180 μl in each well. Ten microlitre spot plate counts were placed on ½ LB agar using a Research Plus multichannel micropippetor (Eppendorf) following cell dilution, and incubated at 37°C overnight. Infection plates were wrapped in cellophane to reduce evaporation of liquid from wells and placed at 30°C in the dark. Plant survivors were counted at 96 h. Plants were identified as “alive” when more than 10% of the plant remained green after 96 h, and plants that displayed >90% loss of green pigmentation were considered dead. Each independent trial consisted of 4-8 replicate infections serially diluted 5 times, and separate overnight cultures were grown for each trial. LD_50_ values represent the average of replicates ± standard error, and LD_50_ values among the strains were compared using Student’s T-tests. LD_50_ values for duckweed and wax moth larval infections were determined according to the protocol described by Randhawa [32], with LD_50_ values derived from each independent trial combined to produce an average and standard error. A sample calculation is shown in Figure S1.

 EPEC strains were grown similarly to Bcc strains but in full-strength LB and infections were performed as described for Bcc, except that plant survival was measured at 7 days instead of 96 h as a result of EPEC-mediated killing having a delayed onset (≥ 5 days) relative to that observed with Bcc strains. Two to four trials were carried out for all strains, and LD_50_ values represent the average of replicates ± standard error. Student’s T-tests were used to compare LD_50_ values among the strains.

### Surface sterilization of infected plants

 Using a sterile inoculating loop, each infected plant was transferred to a well containing 200 μl 8% bleach. A sterile pipette tip was used to submerge the plant for 30 s, and the plant was transferred 3 times into wells containing 200 μl SHS media, each time submerging the plant, to remove all traces of bleach. Plants were left in the third wash well while replicate plants were surface-sterilized, and each plant was then transferred into a microfuge tube containing 25 μl SHS. Using an ethanol-sterilized plastic micropestle (Sigma-Aldrich, St. Louis, MO), plants were homogenized until no trace of plant tissue was visible (usually 30 - 60 s). Twenty microlitres of this homogenate was transferred into 180 μl SHS, serially diluted and spotted onto LB agar to obtain plate counts. To count bacterial survivors of the bleach treatment that could contaminate the invading bacterial numbers, the final wash solutions of each replicate were plated on LB agar. Three independent trials were carried out with four replicates of each sample per trial. 

### Plasposon library screening of *B. cenocepacia* K56-2

 A mutant library carrying random genomic insertions was produced by electroporating *B. cenocepacia* K56-2 with plasposon pTn*Mod*-OTp’ and plating transformants on LB containing μg/ml trimethoprim (LB + Tp), as previously described [33]. Duckweed plants were placed individually into wells of a 96-well plate containing 200 µl SHS. Mutant strains were grown in 96-well plates for 40 h in 200 µl ½ LB + Tp100 at 30°C with shaking at 225 rpm, then ~5 µl from each well were transferred to the plant-containing wells using a sterile 96-pin Multi-Blot Replicator (VP Scientific, San Diego, CA). Inoculated plants were then incubated at 30°C for 4 days, and surviving plants were recorded. Plasposon insertion sites were determined by plasposon rescue, as previously described [34], and sequencing was performed using BigDye Terminator v3.1 (Life Technologies, Carlsbad, CA) with sequencing primers JD28 5’ ori (GGGGAAACGCCTGGTATC) and JD47 3’ Tp (TTTATCCTGTGGCTGC). Virulence genes identified in the mutant screen were submitted for PHYRE2 analysis [35] when homology was not readily available at NCBI. To identify orthologs of the virulence genes among sequenced *Burkholderia* strains, the *Burkholderia* Genome Database (http://www.burkholderia.com/) was consulted.

### Genetic complementation of mutant *46B2*

 To restore the virulence phenotype of mutant 46B2, *bcal1124* was amplified by colony PCR from *B. cenocepacia* K56-2 according to manufacturer’s instructions using TopTaq (Qiagen, Inc., Hilden, Germany) and the following oligonucleotides (Sigma-Aldrich): 1124-F, TTATTACATATGAATAACGTTAATGAAGACCAGG, and 1124-R, ATAAAGCTTTTA**ATGATGGTGATGGTGATGATGGTGATGGTG**TTCATTCTGGTCCTTATCC (restriction sites are underlined, and a 10x polyhistidine tag is shown in bold). The resulting PCR construct and plasmid pSCRhaTc [33] were digested with FastDigest enzymes NdeI and HindIII (Thermo Fisher Scientific, Ltd., Waltham, MA), separated by gel electrophoresis, and purified using GeneJET Gel Extraction Kit (Thermo Fisher Scientific). The products were then ligated using T4 DNA ligase (Promega Corp., Fitchburg, WI) at 16°C overnight. *E. coli* DH5α was transformed with 5 µl of the ligation mixture and transformants were selected on LB + Tc. Plasmids were then extracted and compared by restriction digestion and gel electrophoresis as well as BigDye sequencing. *B. cenocepacia* K56-2 was transformed by electroporation and selection on LB + Tc. Plasmid isolations were carried out using GeneJET plasmid miniprep kit (Thermo Fisher Scientific). 

### Bacteriophage rescue

 Ninety-six well plates were prepared as above, but only 160 μl of SHS was added to each well. For the preliminary trials, 20-μl of a dilution of bacteria corresponding to ~100 × LD_50_ was added to each infection well. Follow-up trials used inocula of ~10^6^ × LD_50_. An additional 20 uL of 4x10^8^ pfu/ml phage stock in sterile mQ H_2_O was added to each infection well. Control trials included uninfected plants with and without phage and infected plants without phage. Each group of six plants infected with a given overnight culture of bacteria was counted as a single trial. 

## Results

### Establishment of duckweed as a model host for the *B. cepacia* complex

**Figure 1:**
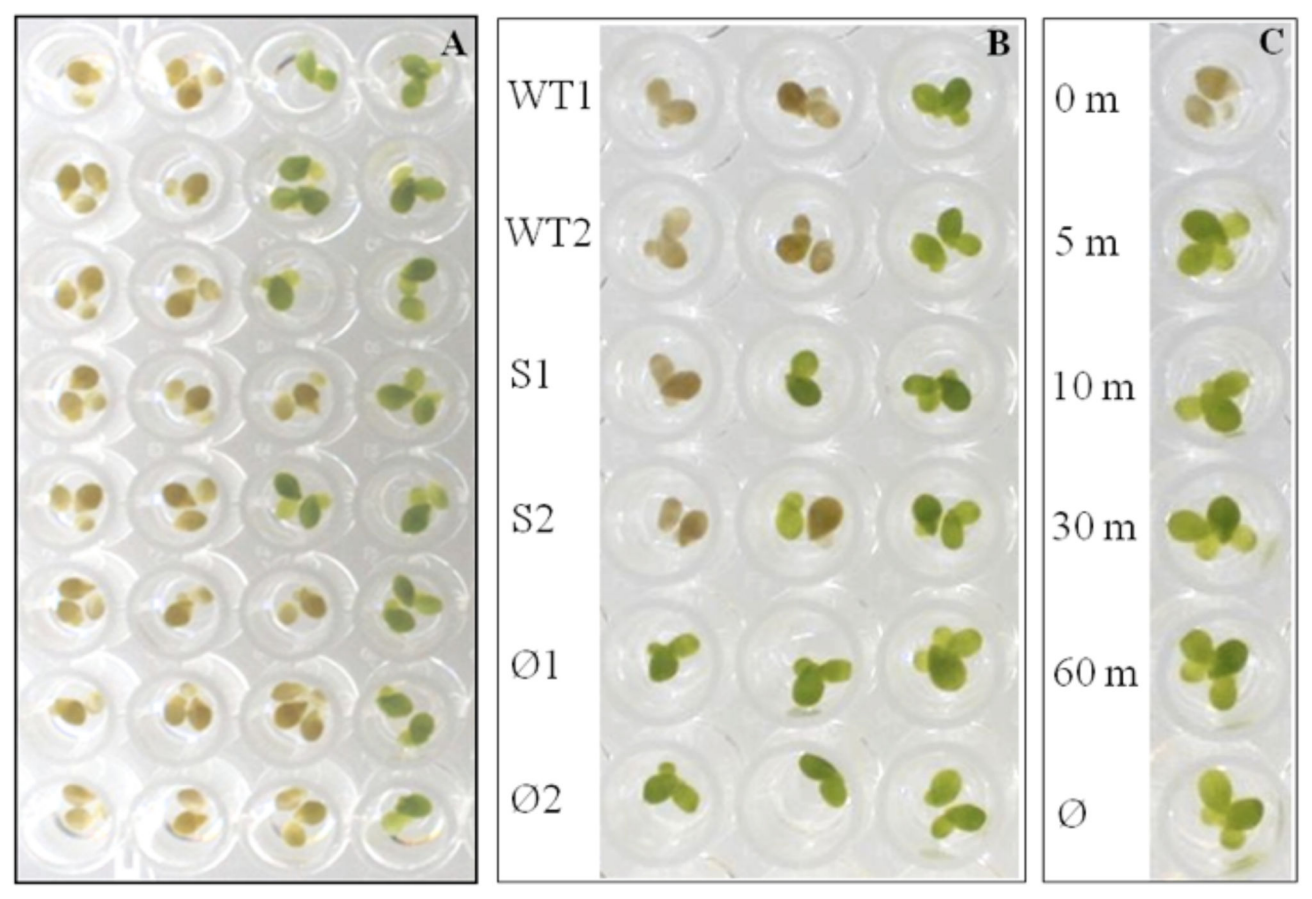
*Lemna* *minor* provides both quantitative and qualitative assessments of bacterial strain lethality. **A**. 50% lethal dose can be determined by assessing plant survival after a given timepoint. An overnight culture of a given strain was washed and inoculated into each well of column 1, then 10-fold serially diluted into each subsequent column using a multichannel pipette. Bacterial counts were generated using a multichannel pipette by spotting 10 μl from each well on agar. For Bcc infections, surviving plants were counted after 96 h. **B**. Supernatants of wild type and *shvR*-deficient *B*. *cenocepacia* affect duckweed at different dilutions. Two 5-day cultures were lyophilized, resuspended in deionized water and 4-fold serially diluted into duckweed-containing wells for each strain: WT, wild type *B*. *cenocepacia* K56-2; S, K56-2 harbouring a Tp^R^ cassette in gene *bcas0225*, also known as *shvR*; Ø, blank SHS media control. Results are shown at 72 h post-inoculation. **C**. Heat-killed *B*. *cenocepacia* K56-2 has no effect on duckweed. A dense (~ 1x10^9^ cfu/ml) K56-2 suspension was incubated at 65°C for different lengths of time (shown in min beside each well) and then inoculated into a plant-containing well. A lack of viable cells from a 5-min incubation onward was shown by spotting the suspensions on LB agar.

To generate an accurate, reproducible 96-well plate-based infection assay, single axenically-grown duckweed plants derived by asexual reproduction from a single plant were placed into individual wells of a 96-well plate containing SHS media and infected with serial dilutions of a *B. cepacia* complex pathogen, *B. cenocepacia* K56-2. Plants began to show signs of morbidity at high doses by 24 h, with Bcc infections reaching completion at 4 days. After this time, surviving plants tended to persist, having resisted the initial infection. Figure 1A shows a typical result at 96 h: none of the plants survive the highest bacterial loads, 50% of the plants survive the third dilution, and all of the plants survive the fourth dilution. To determine whether the plant killing effect in part was due to soluble factors released from the bacteria, cell-free bacterial filtrates were added to the plants. Whereas wild type K56-2 supernatants killed plants at 16-fold dilution (i.e., 0.18 mg/ml protein), supernatants of the *shvR* mutant killed plants only at 4-fold dilution (i.e., 0.775 mg/ml protein; Figure 1B), though partial plant damage was still observed at 16-fold dilution with the *shvR* supernatant. Boiling the supernatants for 10 min had no effect on their toxicity, indicating that the toxic compound is heat-stable. Inoculation of plants with ~10^9^ cfu /ml heat-killed bacteria had no effect on the plants (Figure 1C). Zhang et al. [31] demonstrated that infection with *S. aureus* RN4220 resulted in dramatic drops in both plant fresh weight and chlorophyll concentration, but because these Bcc infections were performed in the absence of light there was minimal plant growth following the infection periods, and therefore single plants showed little difference in fresh weight following infection. However, the bleaching effect we observed in the dead plants was also the result of chlorophyll degradation, as measured following ethanol extraction of infected plants (data not shown). Although the use of a qualitative visual endpoint determination in the current method results in a simple and inexpensive assay, the sensitivity of this assay could potentially be improved by automating the quantification of the extent of plant morbidity and chlorophyll degradation by a method similar to that described by Schikora et al. [36], who produced a pixellation algorithm to monitor *Salmonella* Typhimurium infection of *Arabidopsis*.

### Correlation between Bcc virulence in plant and insect models

**Figure 2:**
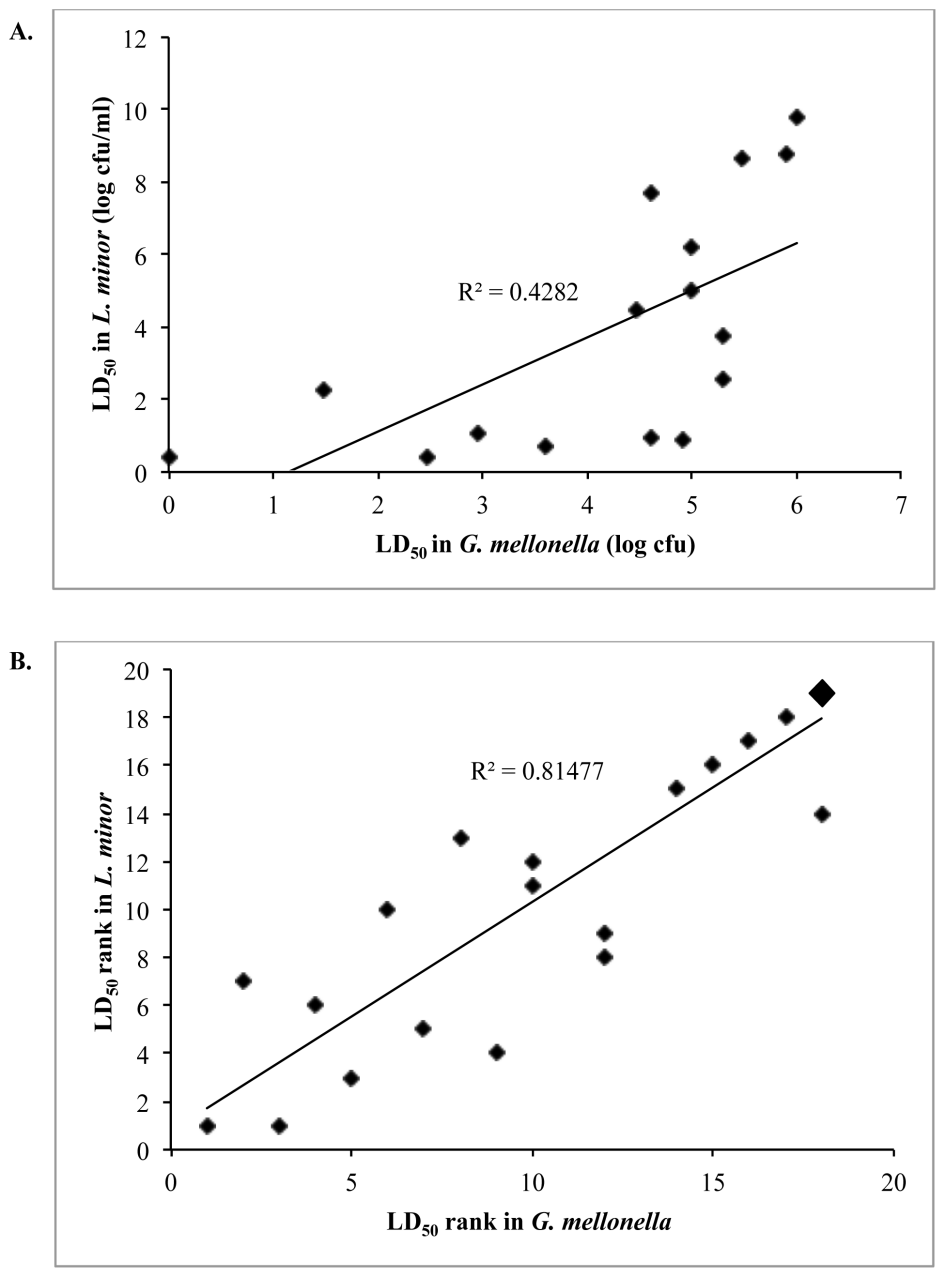
The virulence of Bcc strains in duckweed correlates with their virulence in wax moth larvae. **A**. Each point represents the LD_50_ of a Bcc strain in duckweed determined from the compiled data of 2-6 independent trials plotted against LD_50_ values determined in wax moth larvae [13]. **B**. LD_50_ values for all strains in both infection models placed in order of rank. The large point represents four *B*. *multivorans* strains (C7322, C3430, C5393, and PC249) and one *B*. *dolosa* strain (AU0645) for which bacterial loads high enough for killing were not attained. NB. all strains identified by Seed and Dennis [13] to be avirulent in the larva model were also avirulent against duckweed, except for *B*. *multivorans* C5568 with an LD_50_ of 1.06x10^8^ cfu/ml.

The virulence of the different Bcc species in duckweed is generally consistent with the findings of Bernier et al. [8], in which alfalfa seedlings were used as a model infection host. Virulent species included *Burkholderia cepacia, B. cenocepacia, Burkholderia vietnamiensis, Burkholderia dolosa* and *Burkholderia ambifaria*, whereas *Burkholderia multivorans* and *Burkholderia stabilis* were relatively avirulent. One exception to this is *B. ambifaria* Cep0996, which shows low virulence against duckweed, whereas *B. ambifaria* AMMD is highly virulent (LD_50_ = 2.5 cfu/ml; not shown in Figure 2 as it was not included in the analysis of Seed and Dennis [13]). We extended this analysis to place *Burkholderia pyrrocinia* and *Burkholderia anthina* in the virulent and avirulent categories, respectively, and provide an alternative quantitative approach by which to directly compare strain virulence among different infection models. Neither our results nor those of Bernier et al. [8] are consistent with the findings of Yohalem and Lorbeer [27], who found that Bcc strains of clinical origin were unable to cause maceration upon onion tissue inoculation, whereas many environmental strains, particularly isolates from onion rot, were highly pathogenic. This discrepancy could be a result of the inoculation method; whereas the alfalfa seedlings and duckweed plants are left intact during incubation with the bacteria, the onion maceration model involves cutting the onion open and inoculating bacteria into the damaged tissue. Alternatively, this discrepancy could simply be an indication of common Bcc virulence factors at play for clinical, duckweed, and alfalfa infections that are not significant factors in the onion model. 

 A useful quantitative gauge of Bcc virulence is the *G. mellonella* (Greater wax moth) larval infection model, introduced for the Bcc by Seed and Dennis [13]. This study established LD_50_ values for a panel of Bcc strains representing 9 of the 17 established Bcc species. *B. cepacia* and *B. pyrrocinia* strains were the most virulent in this model, whereas *B. dolosa, B. ambifaria* and *B. multivorans* were the least virulent. It was noticed early in our study that several of the most virulent strains in wax moth larvae were also highly virulent against duckweed. Therefore, we investigated the relationship between LD_50_ values of these representative Bcc strains in both models. Consistent with our early findings, the most virulent strains against wax moth larvae were also the most virulent against duckweed, and the same trend was observed for the least virulent strains (Figure 2A). Although a weak correlation was found between the raw LD_50_ values in duckweed and larvae (R^2^ = 0.43), transforming the LD_50_ values into rank format yielded much stronger agreement (R^2^ = 0.81), showing that the relative virulence of members of the Bcc are consistent between the two models (Figure 2B).

### Extension of the duckweed model to enteropathogenic *Escherichia coli*

**Figure 3:**
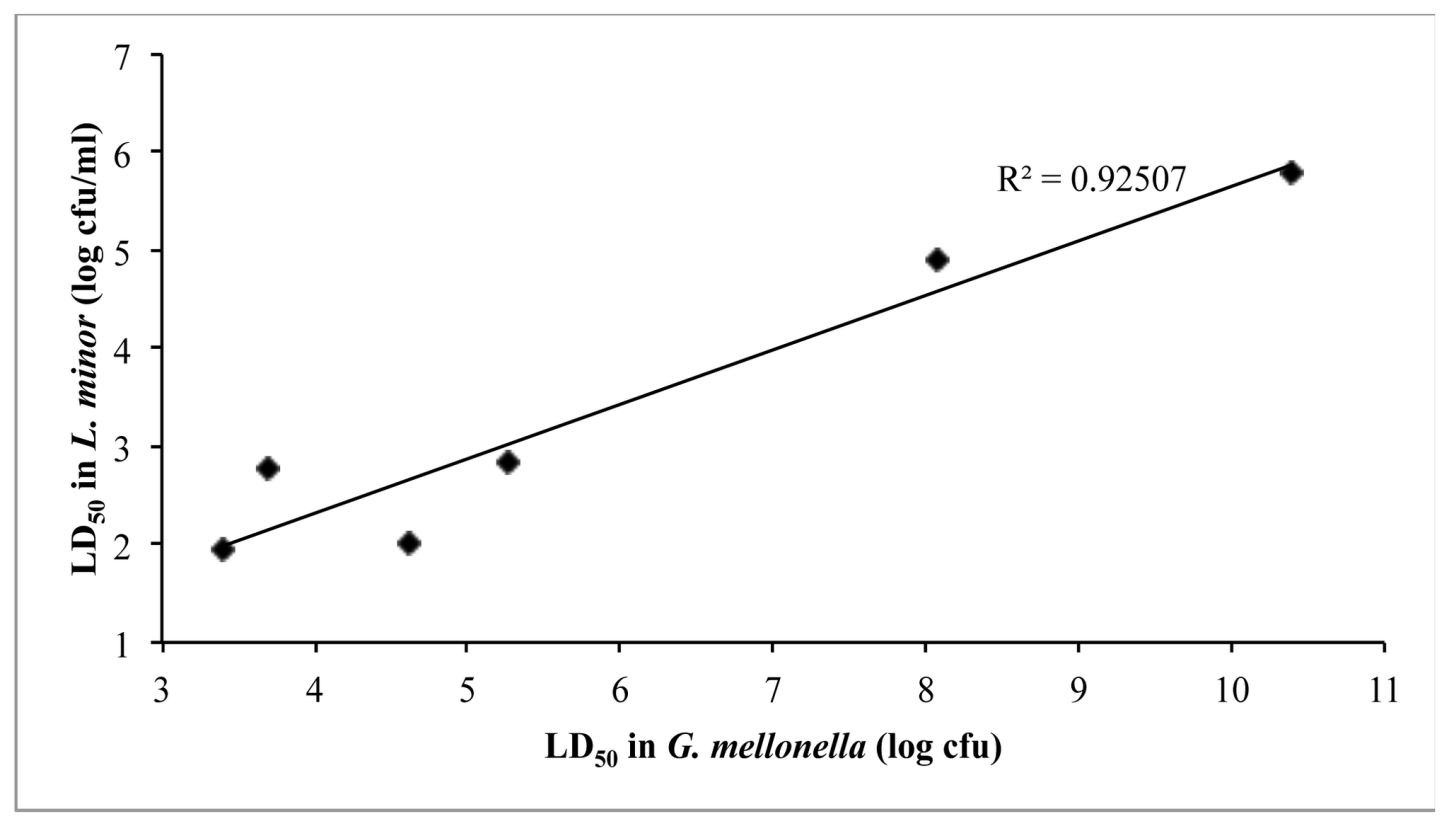
The virulence of EPEC strains in duckweed correlates with their virulence in wax moth larvae. Wild type EPEC and five mutant strains were inoculated into wells containing duckweed and left to incubate at 30°C for 7 days. Each point represents the LD_50_ determined in duckweed from the compiled data of 2-4 independent trials plotted against values determined in wax moth larvae by Leuko and Raivio [37].

The same approach used to analyze the Bcc virulence was applied to other bacterial pathogens with LD_50_ values established in the wax moth larvae. Of the assayed bacteria, *Acinetobacter baumanii, Campylobacter jejuni*, and enteropathogenic *E. coli* (EPEC), only EPEC showed virulence against duckweed. We also tested the tomato pathogen *Ralstonia solanacearum* against duckweed, and even after plant wounding or piercing, the bacteria were unable to establish an infection. EPEC virulence was expected, as Zhang et al. [31] observed virulence of enterohaemorrhagic *E. coli* (EHEC) but avirulence of lab strain DH5α against duckweed. We established LD_50_ values for wild type EPEC versus five isogenic mutants deficient in bundle forming pilus (bfp), a type III secretion system (*escN*; T3SS), the EAF virulence plasmid (JPN15; deficient in T3SS activation and BFP), the CpxR response regulator (*cpxR*; inactive Cpx stress response pathway), and CpxA phosphatase activity (cpxA24* that results in a constitutively activated Cpx pathway). The LD_50_ values obtained for all six strains reflect a trend observed by Leuko and Raivio [37] using the wax moth larva model (Figure 3). 

### Random mutant screening reveals novel putative virulence genes in *B. cenocepacia* K56-2

 To expand the utility of this model system, we performed a high-throughput mutant screening of *B. cenocepacia* K56-2. Of 5,980 mutants screened, we uncovered 12 with increased LD_50_, including 7 with LD_50_ increases of between 5- and 10^7^-fold. The mutants, their determined LD_50_ values, and brief descriptions of their disrupted genes are shown in Table 2. Only two mutations, to the *afc* gene cluster and the *afc*-regulating LysR homolog encoded by BCAS0225 / *shvR* (11G10 and 50D9, respectively), have been previously shown to cause defects in virulence towards plants; it was also shown that ShvR regulates over 1,000 genes and upregulates expression of protease, lipase and type II secretion [38,39]. In addition, whereas a *shvR* mutation was previously shown to have no impact on virulence in invertebrate models [30], the mutation reduced virulence in the rat agar bead model. Further research has shown that this gene cluster gives rise to a membrane-associated antifungal lipopeptide and its production decreases membrane permeability and alters lipid metabolism in K56-2 [40]. Mutants 11G10 and 50D9 had the greatest increases in LD_50_ and were unable to kill duckweed at even the highest assayed titres. 

**Table 2:** Attenuated *B. cenocepacia* K56-2 plasposon mutants identified against individual duckweed plants.

**Strain**	**LD_50_ cfu/mL +/- SE^a^**	**Plasposon insertion locus^b^**	**Characteristics**	**Prevalence among sequenced *Burkholderia* species**
WT	1.2x10^1^ +/- 7.0x10°	n/a	n/a	
11G10	>1x10^9^	*bcas0210*: Antifungal compound synthase (AFC) cluster	AFC cluster genes influence biofilm formation, lipid metabolism, swarming motility and antifungal activity [40]	*B. cenocepacia, B. ambifaria, B. lata, B. pseudomallei, B. mallei*
12C9	2.1x10^5^ +/- 4.6x10^4^	*bcal0311*: HisG (ATP phosphoribosyltransferase)	Part of histidine biosynthesis cluster; upregulated 2-fold during chronic CF infection [46]	Ubiquitous
16A11	~1x10^6^	*bcal2159*	Hypothetical α/β barrel domain-containing protein; downstream from SuhBBc [42]	*B. cenocepacia, B. lata, B. multivorans, B. gladioli, B. xenovorans*
32B11	1.9x10^3^ +/- 1.5x10^3^	Intergenic between *bcal0549* and *bcal0550*		n/a
42H4	3.5x10^4^ +/- 1.7x10^4^	n.d.		
46B2	7.7x10^1^ +/- 1.8x10^1^	*bcal1124*: Hypothetical protein in BcenGI5 genomic island	Putative viral origin of replication-binding protein	*B. cenocepacia, B. pseudomallei*
50D9	>2.6x10^8^	*bcas0225*: ShvR	LysR-like protein regulates AFC cluster, quorum sensing, protein secretion, protease and lipase production, and virulence [8,39,46]	*B. cenocepacia, B. ambifaria, B. lata, B. gladioli*
51H6	~1x10^3^	*bcas0134*: LysR-like regulator	Putative LysR-like regulator, regulated by BDSF signalling [41]	Limited to *B. cenocepacia* ET-12 epidemic lineage
62F12	~5x10^5^	*bcal0870*	Putative oxidoreductase, identified as an essential gene in *B. cenocepacia* H111 [43]	Ubiquitous

a S.E., standard error of the mean, included where applicable

b n.d., not determined.

 Because mutation of *shvR* or the adjacent AFC genes caused a seemingly full reduction in virulence, it was surprising that a series of apparently unrelated genes also emerged from our mutant screening. Uncharacterized genes whose disruption caused decreases in virulence against duckweed include: *bcas0134* (mutant 51H6), encoding a novel *lysR* regulator negatively regulated by the recently-characterized BDSF quorum sensing signalling pathway [41]; *bcal1124* (mutant 46B2), a hypothetical protein encoded on a genomic island; *bcal0870* (mutant 62F12), encoding a putative oxidoreductase; and *bcal2159* (mutant 16A11), which is located near the recently characterized *suhB* gene, *bcal2157*. Elimination of SuhB_Bc_, which encodes an inositol phosphatase predicted to play a role in intracellular signalling, was shown to abolish or reduce type II and type VI protein secretion, biofilm formation, motility and polymyxin resistance, and causing a 2-fold decrease in growth rate [42]. Interestingly, *bcal0870* was predicted to be an essential gene by a recent *in silico* study that produced a core genome for the order *Burkholderiales* comprising 649 genes [43]. Given our finding that cell viability is retained after *bcal0870* mutation, this finding is likely a false prediction. To date, any relationship between these genes and the ShvR/AFC cluster has not been ascertained. 

**Figure 4:**
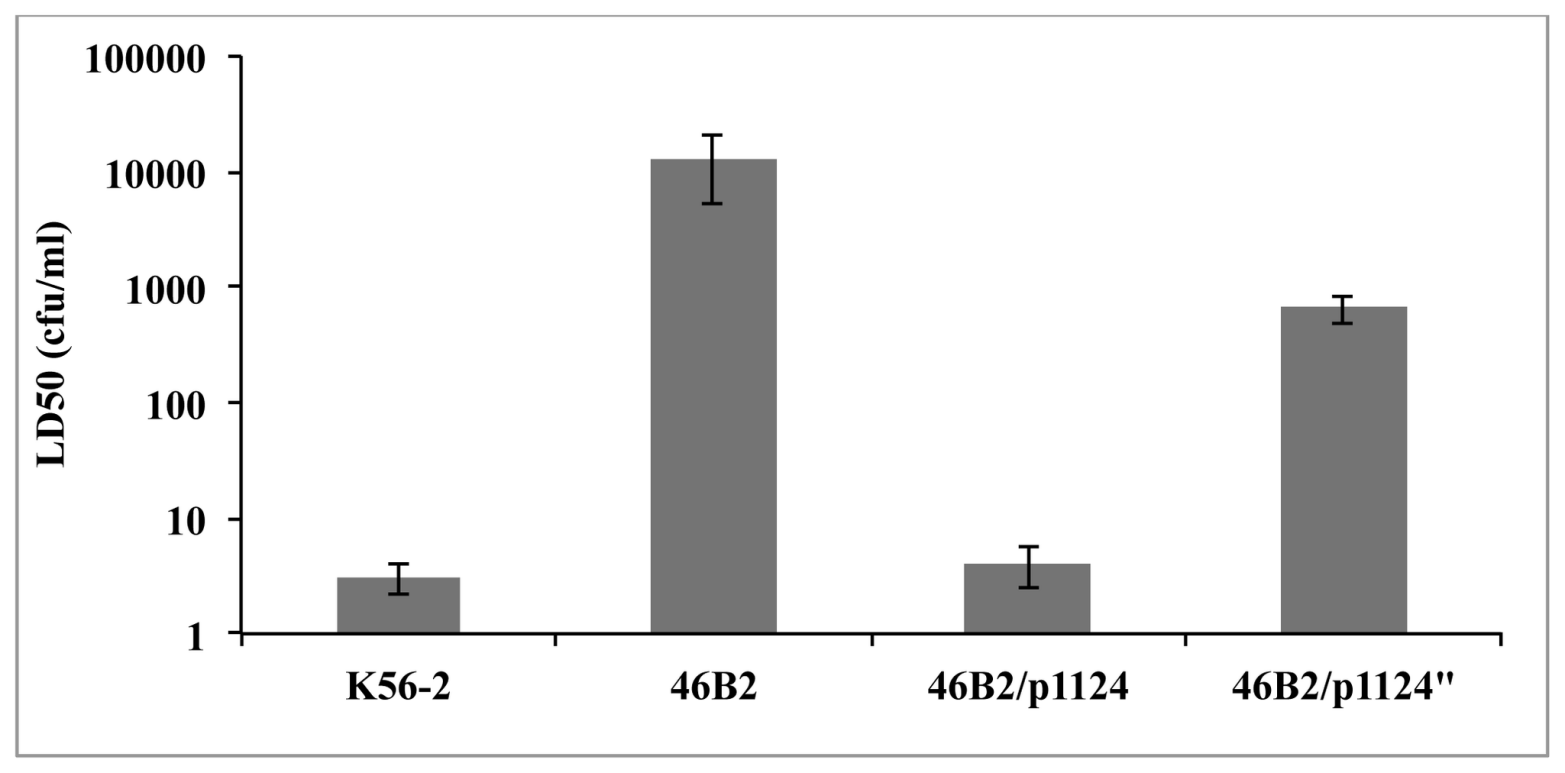
Complementation of mutant 46B2 with *bcal1124*, but not *bcal1124* with point mutations at R204H and E336G, results in restoration of plant killing. Infection experiments were performed as described above following overnight growth of all strains (K56-2/pSCRhaTc, 46B2/pSCRhaTc, 46B2/p*1124*, 46B2/*p1124*”) in ½ LB + Tc100 + 0.02% w/v rhamnose. Infections omitted antibiotics but included 0.02% rhamnose to continue induction of the pSCRha promoter.

 Although mutant 46B2, which carries a plasposon insertion in *bcal1124*, had the smallest virulence attenuation among the recovered mutants, it also demonstrated an overgrowth phenotype that produced visible turbidity in the infection wells (Figure S2). While the LD_50_ reflects the starting bacterial density requirement for plant mortality, 46B2 in fact produces a much greater population than the parental strain when incubated with plants. Therefore, this protein was subjected to further investigation. A BLASTP search revealed that homologs of *bcal1124* are limited to *B. cenocepacia* among the Bcc, but also appear in other bacterial pathogens including *Burkholderia pseudomallei, P. aeruginosa*, and *A. baumannii*. Bioinformatic analysis of Bcal1124 suggests that it contains a DNA binding domain, as suggested by its strong structural resemblance to a replication-binding protein of viral origin as predicted by PHYRE2 analysis [35]. To confirm the importance of *bcal1124* as a virulence determinant, mutant 46B2 was complemented using plasmid pSCRhaB2 modified with a tetracycline resistance cassette. Two variations of the complementation construct were used to transform 46B2::p*1124*, one containing the wild type *bcal1124*, and p*1124*”, which contains point mutations at R204H and E336G that were produced during PCR amplification. Both versions are tagged at their N-termini with 10x polyhistidine tags. Whole cell lysates were probed by immunoblot and Bcal1124 was detected at approximately 55 kDa (data not shown), slightly larger than the expected 47 kDa predicted for Bcal1124^His^. The strains were tested for virulence in duckweed, and whereas 46B2/p*1124* demonstrated full restoration of virulence, 46B2/p*1124*” showed only a partial restoration of virulence (Figure 4), suggesting a deleterious effect on Bcal1124 by either one or both point mutations. Interestingly, the R204H mutation resides near the DNA binding domain predicted by PHYRE2 analysis (amino acids 212-266).

### Dynamics of bacteriophage rescue of plants from *B. cenocepacia* infection

**Figure 5:**
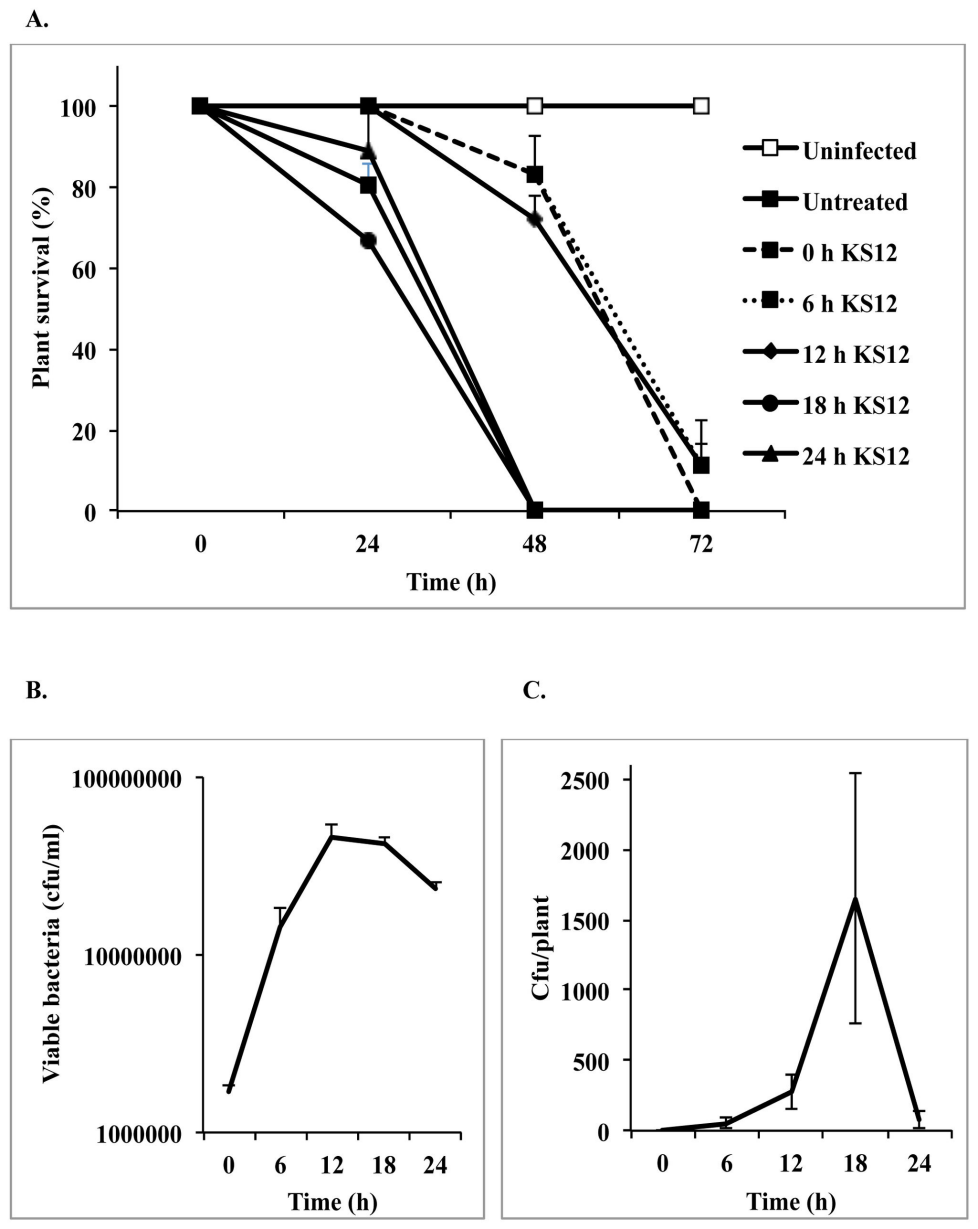
*B*. *cenocepacia* infection of duckweed is alleviated by bacteriophage treatment up to 12 h but not after 18 h. **A**. *B*. *cenocepacia* K56-2 was inoculated into plant-containing wells at 2x10^6^ cfu/ml, with 4x10^8^ pfu/ml of phage KS12 (i.e. MOI = 200) applied at 0, 6, 12, 18, and 24 h. Results shown are averages of 3 independent trials using 6 plants per trial +/- SE. “Untreated” refers to both phage-treated and mock-treated plants, as both showed 100% survival. **B**. Bacterial counts from media surrounding plants during the infections (not phage-treated). Results shown are averages of 3 independent trials performed in triplicate +/- SE. **C**. Bacterial counts from crushed surface-sterilized plants. Results shown are averages of 3 independent trials +/- SE, with four replicates of each sample per trial.

We sought to determine whether bacteriophage treatment could rescue duckweed plants from *B. cenocepacia* K56-2 infection, and if so, whether a timeline exists for effective treatment. A preliminary experiment suggested that when phage were added at 4 h post-infection, plants showed considerably higher survival rates than when phage were added at 24 h post-infection (Figure S3). Therefore, we investigated the phenomenon at a finer scale, applying phage every 6 h up to 24 h. We used high bacterial loads in these experiments to investigate tissue invasion as a possible Bcc escape strategy. The results suggest that after 12 h, no difference exists between the survival of treated and untreated plants (Figure 5A), despite the fact that bacterial counts in the medium surrounding the plants reach their peak at 12 h (Figure 5B). Bacterial invasion of plant tissue was measured by surface-sterilization of the plants with bleach, followed by homogenization of the plants and viable plate counts of crushed plant matter. Bacteria were observed at high numbers inside the plants by 18 h, increasing approximately 10-fold by 24 h (Figure 5C), suggesting that K56-2 may escape phage treatment by invading plant tissues. The reduction in bacterial counts at 24 h is suspected to be the result of bleach penetrating damaged plant tissues or toxic HR activity within the plant tissues as a result of infection [44]. 

## Discussion

 The usefulness of duckweed as an alternative infection model for pathogenic bacteria was confirmed when we established a correlation between Bcc virulence in plant and insect infection models. We observed that several of the most virulent strains in wax moth larvae infection model [13] were also highly virulent against duckweed. Examining the relationship between LD_50_ values of representative Bcc strains in both models, we discovered a trend showing the most virulent Bcc strains against wax moth larvae were also the most virulent against duckweed, and a similar trend was observed for the least virulent strains (Figure 2A). This relatively weak correlation between LD_50_ raw scores in duckweed and wax moth larvae (R^2^ = 0.43) was strengthened by rank ordering the LD_50_ values of each strain (R^2^ = 0.81), although both analyses demonstrate a general virulence trend for species of the Bcc (Figure 2B). Therefore, it is anticipated that conserved virulence factors confer similar virulence levels across the two infection models, as has been observed for other pathogenic bacteria. For example, *P. aeruginosa*’s multihost pathogenicity was used to identify a several genes previously found to encode gene products involved in *Pseudomonas syringae* plant infections [45]. A similar situation is beginning to emerge for *B. cenocepacia* where the LysR-like regulator *shvR*, initially identified as a major virulence factor in alfalfa [38], has now been shown to control a regulon of over 1,000 genes that includes genes for quorum sensing and other universal virulence factors [46].. 

 Similarly, we established a trend for LD_50_ values of wild type EPEC versus five isogenic mutants deficient in several well-characterized virulence factors, including bundle-forming pili, the *cpx* stress response regulators, and a type III secretion system. The LD_50_ values obtained for all six strains reflect a trend observed by Leuko and Raivio [37] using the wax moth larva model. We observed that the elimination of the EAF virulence plasmid causes a larger increase in LD_50_ than either the elimination of either type III secretion, which depends on the plasmid-encoded regulators PerA, PerB and PerC for transcriptional activation of the T3SS, or the bundle-forming pilus, which is genetically encoded by the plasmid. This finding suggests that the EAF plasmid potentially supplies an additional virulence factor other than the well-characterized T3SS and BFP [47,48]. T-tests reveal that only strain JPN15 (lacking the EAF plasmid) differs significantly from wild type EPEC (p=0.06).

The mechanism of plant mortality in these infections was not determined. Although plants lack roaming defender cells and adaptive immune systems, their innate immune systems comprise two interacting pathways to defend against bacterial pathogens: effector-triggered immunity and pathogen associated molecular pattern (PAMP)-triggered immunity. Built into these oscillating pathways is the hypersensitive response (HR), which is associated with reactive oxygen species (ROS) production and localized cell death in the presence of an overwhelming infection [49]. In Bcc infection of duckweed, our data suggest one of two possibilities: either that the observed plant death occurs in a HR-independent manner mediated primarily by invasion and/or toxin production, or that Bcc strains engage the HR to varying extents and the total chlorophyll bleaching that leaves the plants brown is simply an extreme HR. In the first scenario, it was hypothesized that the product of the *afc* gene cluster, a toxin previously characterized in both *B. cepacia* BC11 [50] and *B. cenocepacia* K56-2 [39], could be primarily responsible for this effect, so exported toxin production was tested by lyophilizing and concentrating culture supernatants from both wild type *B. cenocepacia* K56-2 and an isogenic *shvR* mutant obtained during the plasposon mutant screening. Whereas wild type K56-2 supernatants killed plants at 16-fold dilution (i.e., 0.18 mg/ml protein), supernatants of the *shvR* mutant killed plants only at 4-fold dilution (i.e., 0.775 mg/ml protein; Figure 1B), though partial plant damage was observed at the 16-fold dilution in the *shvR* concentrate. This result demonstrates that K56-2 kills plants in part through exported toxin production but that additional factors beyond the *afc* cluster adjacent to *shvR* may also affect the plants. In the second scenario, extreme HR by the plants would be caused by plant responses to PAMPs found on bacterial cell surfaces. However, inoculation of plants with ~10^9^ cfu /ml heat-killed bacteria had no effect on the plants, indicating that the observed plant killing was not HR-mediated. Combined, we can conclude that *B. cenocepacia* K56-2 kills duckweed through a combination of exported toxin and contact-dependent mechanisms, but likely does not activate a lethal hypersensitive response through surface appendages. 

 The isolation of attenuated *B. cenocepacia* K56-2 plasposon mutants from a screen against individual duckweed plants revealed a diversity of novel putative virulence genes responsible for plant morbidity. In addition to validating this infection model by identifying known Bcc virulence factors (including *shvR* and the AFC toxin) isolated using different methods and characterized in previous studies [38-40,46], we also discovered the genes for several new and uncharacterized Bcc putative virulence factors. These genes include *bcas0134*, encoding a novel *lysR*-family regulator regulated by the recently-characterized BDSF quorum sensing signalling pathway [41], *bcal1124*, a hypothetical protein encoded on genomic island BcenGI5, *bcal0870*, encoding a putative oxidoreductase, and *bcal2159*, which is located near the recently characterized *suhB* virulence gene, *bcal2157* [42]. All of these genes may have some potential importance in Bcc pathogenicity, although the changes in LD_50_ relative to wildtype in the duckweed infection model were highest for the *shvR* and the *afc* mutants. Because mutating *shvR* and associated *afc* genes causes complete attenuation of virulence, it follows that other virulence pathways may be regulated or modified by *shvR* and possibly by the compound produced by the *afc* genes. Further characterization of these genes and gene products will help to better delineate the specific and universal virulence mechanisms of the Bcc. 

Using the duckweed infection model, bacteriophage rescue of *B. cenocepacia*-infected plants was shown to depend on time of treatment post-infection, with escape into plant tissues demonstrated as a possible mechanism of bacterial survival (Figure 5). An alternative explanation for the inability of phage to rescue plants beyond 12 h is that a bacterial toxin is released at high enough doses to enact plant death after this timepoint. This possibility is raised by the result shown in Figure 1B, where plant death is brought about by an exported soluble toxin likely under control of the *shvR* regulator. The possibility therefore remains that toxin production early in the co-incubation of bacteria with plants is the cause of the inability of phage to rescue plants after 12 h, and requires further investigation. 

Overall, the results of this study present the development of a new alternative infection model host for the *Burkholderia cepacia* complex and other potentially pathogenic bacteria that is inexpensive, reliable, rapid and easy to manipulate. As the Bcc relatives *Burkholderia glumae* and *Burkholderia gladioli* emerge as major pathogens of rice crops, duckweed could represent a useful model for the delineation of their virulence pathways and identification of inhibitory factors. Since rice plants are most vulnerable to bacterial panicle blight during flowering [51], duckweed would make an ideal candidate for infection, as its flowering phase can be induced by modifying the light cycle. As well, other pathogens of monocotyledon crops (i.e., grains) could potentially be investigated using common duckweed as an alternative infection model. 

## Supporting Information

Figure S1 **Sample data for determination of *B. vietnamiensis* DBO1 LD_50_.** Data from individual trials were plotted and individual trendline equations were obtained to solve for y = 5 (i.e. 50% survival in probit format). The derived x value was then converted from log(cfu/ml) into cfu/ml. Data points falling between 0 and 100% survival plus one point each at 0% and 100% survival were included, where available. The method utilized was adapted from Randhawa [32]. Trendline equations were as follows: Trial 1, y = -0.5091x + 6.2969; Trial 2, y = -0.456x + 6.3832; Trial 3, y = -0.689x + 5.5508; Trial 4, y = -0.6051x + 6.0205.
(TIF)

Figure S2 **Overgrowth phenotype of mutant 46B2 is partially complemented by constitutive expression of *bcal1124intrans*.** Optical density of 150 µl samples taken from wells of each strain (K56-2/pSCRhaTc, 46B2/pSCRhaTc, 46B2/p*1124*, 46B2/*p1124*”) co-incubated with duckweed. Results shown are the averages of 8 biological replicates +/- SE. *p < 0.01.
(TIF)

Figure S3 **Bacteriophage treatment of *B. cenocepacia* K56-2 infection of duckweed.** Bacteria were inoculated into plant-containing wells at 10^2^ cfu/ml, and at 4 h and 24 h phage KS12 was added at the multiplicities of infection (MOI) shown above the graphs. Bars represent averages of 3 independent trials using 6 plants per trial +/- SD. “Untreated” refers to both phage-treated and mock-treated plants, since both showed 100% survival.
(TIF)
