# Supplementary figures and images for: Common Duckweed (Lemna minor) Is a Versatile High-Throughput Infection Model For the Burkholderia cepacia Complex and Other Pathogenic Bacteria
Source: PLoS One. 2013 Nov 6;8(11):e80102. doi: 10.1371/journal.pone.0080102 (PMC3819297; doi:10.1371/journal.pone.0080102)

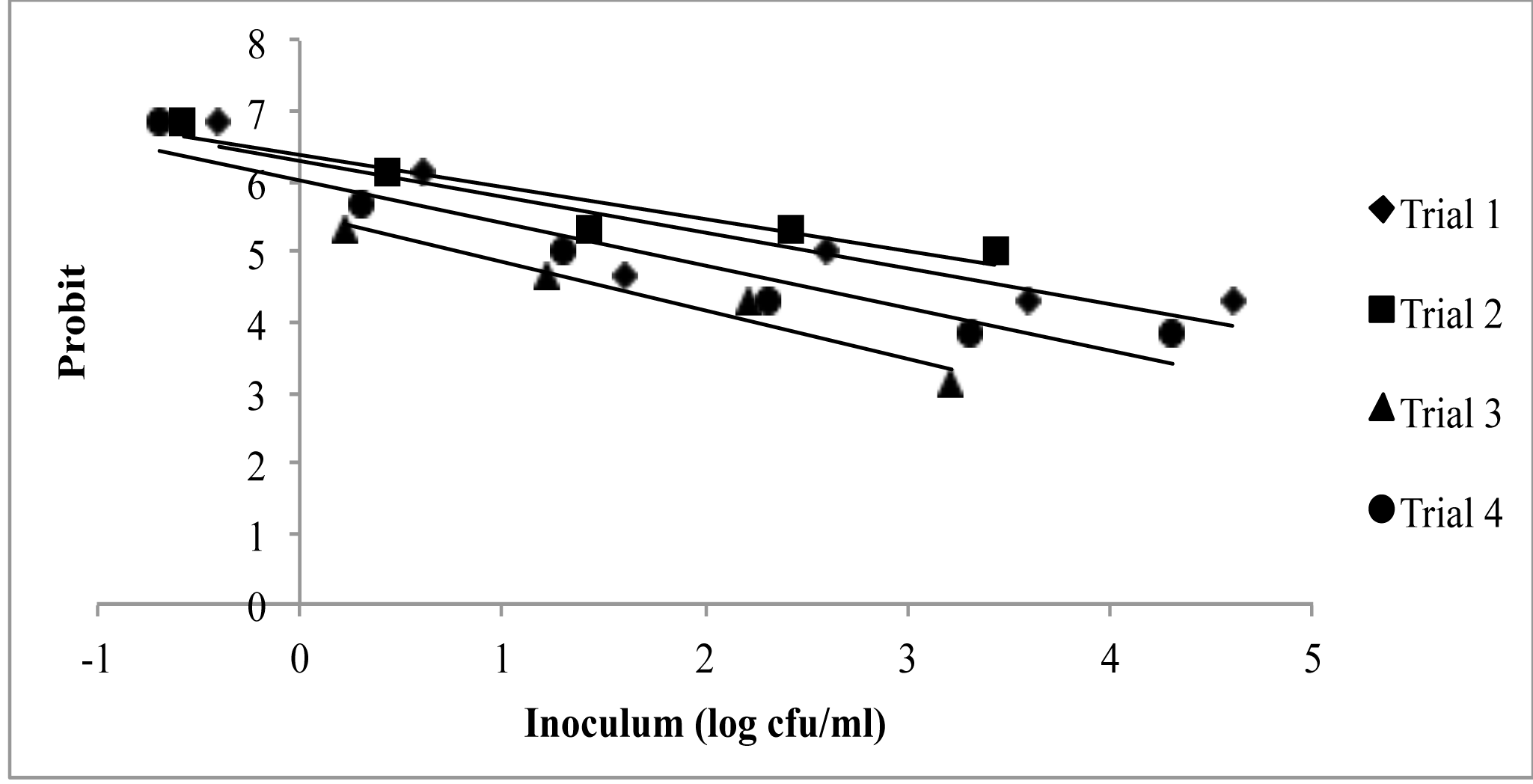

Supplement: Figure S1 — Sample data for determination of B. vietnamiensis DBO1 LD50. Data from individual trials were plotted and individual trendline equations were obtained to solve for y = 5 (i.e. 50% survival in probit format). The derived x value was then converted from log(cfu/ml) into cfu/ml. Data points falling between 0 and 100% survival plus one point each at 0% and 100% survival were included, where available. The method utilized was adapted from Randhawa [32]. Trendline equations were as follows: Trial 1, y = -0.5091x + 6.2969; Trial 2, y = -0.456x + 6.3832; Trial 3, y = -0.689x + 5.5508; Trial 4, y = -0.6051x + 6.0205. (TIF) [file pone.0080102.s001.tif]

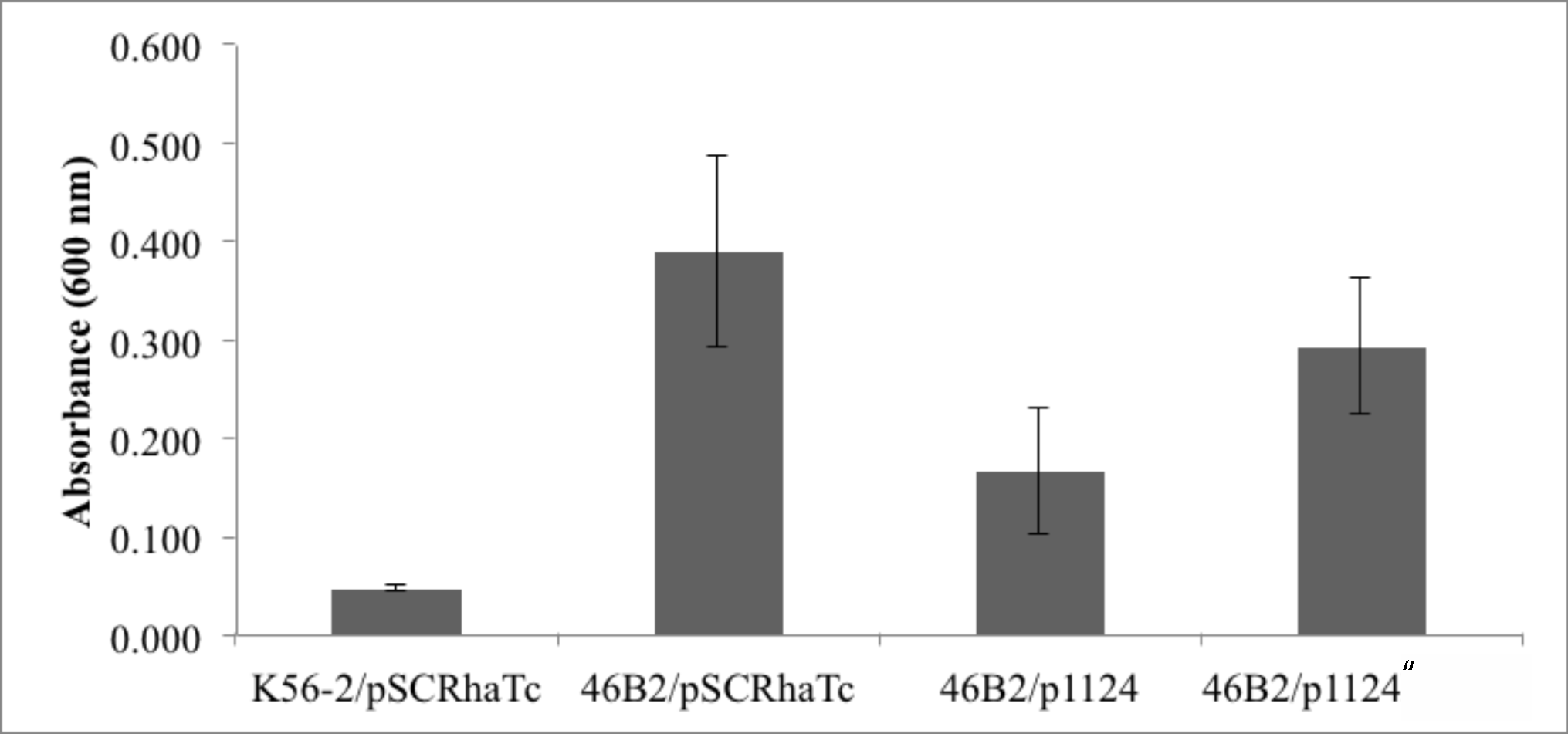

Supplement: Figure S2 — Overgrowth phenotype of mutant 46B2 is partially complemented by constitutive expression of bcal1124intrans. Optical density of 150 µl samples taken from wells of each strain (K56-2/pSCRhaTc, 46B2/pSCRhaTc, 46B2/p1124, 46B2/p1124”) co-incubated with duckweed. Results shown are the averages of 8 biological replicates +/- SE. *p < 0.01. (TIF) [file pone.0080102.s002.tif]

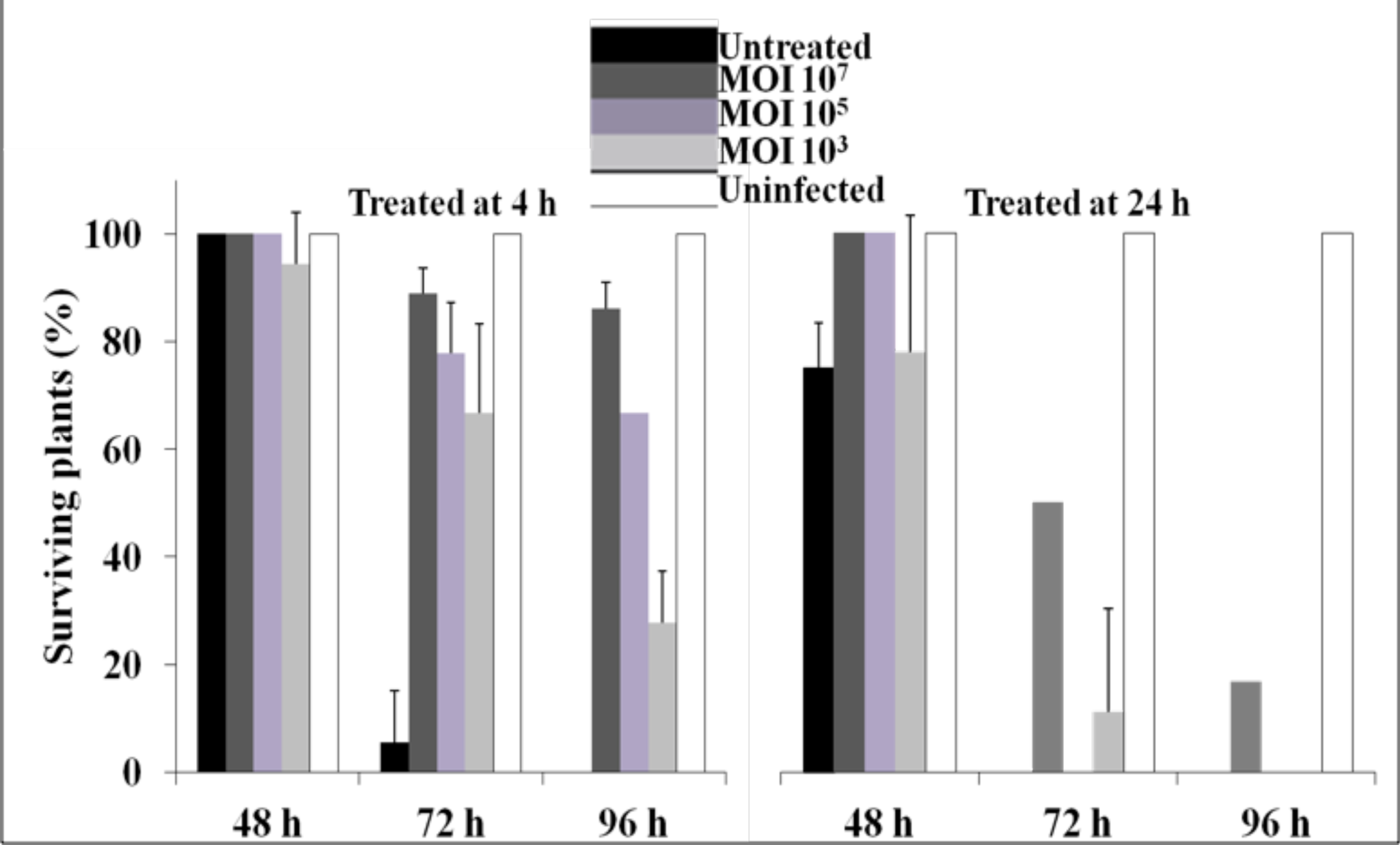

Supplement: Figure S3 — Bacteriophage treatment of B. cenocepacia K56-2 infection of duckweed. Bacteria were inoculated into plant-containing wells at 102 cfu/ml, and at 4 h and 24 h phage KS12 was added at the multiplicities of infection (MOI) shown above the graphs. Bars represent averages of 3 independent trials using 6 plants per trial +/- SD. “Untreated” refers to both phage-treated and mock-treated plants, since both showed 100% survival. (TIF) [file pone.0080102.s003.tif]
